# Problems with equating thermal preference with ‘emotional fever’ and sentience: comment on ‘Fish can show emotional fever: stress-induced hyperthermia in zebrafish’ by Rey *et al*. (2015)

**DOI:** 10.1098/rspb.2016.0681

**Published:** 2017-01-25

**Authors:** B. Key, R. Arlinghaus, H. I. Browman, S. J. Cooke, I. G. Cowx, B. K. Diggles, J. D. Rose, W. Sawynok, A. Schwab, A. B. Skiftesvik, E. D. Stevens, C. A. Watson

**Affiliations:** 1 Brain Growth and Regeneration Lab, School of Biomedical Sciences, The University of Queensland, Queensland 4072, Australia; 2 Dept Biology and Ecology of Fishes, Leibniz-Institute of Freshwater Ecology and Inland Fisheries & Humboldt-Universität zu Berlin, 12587 Berlin, Germany; 3 Institute of Marine Research, 5817 Storebø, Norway; 4 Dept Biology, Carleton University, Ottawa, Canada K1S 5B6; 5 Hull International Fisheries Institute, University of Hull, Hull, HU6 7RX, UK; 6 DigsFish Services, Banksia Beach, Queensland 4507, Australia; 7 Dept Zoology and Physiology, University of Wyoming, Laramie, WY 82071, USA; 8 Infofish Australia, Frenchville, QLD 4701, Australia; 9 Schwab & Sohn, 3507 Biglen, Switzerland; 10 Biomed Sci, Atlantic Veterinary College, Charlottetown, Canada C1A 4P3; 11 Tropical Aquaculture Laboratory, University of Florida, Gainesville, FL 32611, USA

Rey *et al*. [1] report that zebrafish captured with a net and held for 15 min at a water temperature of 27°C exhibited a subsequent preference to swim in water temperatures of 28.75 ± 0.27°C and higher for the next 4 h, compared with control fish that were neither captured nor held in nets. They report that approximately 25% more net-confined fish resided in areas with water temperatures of 29°C or higher (compared with controls; their fig. 2). Based on these results, they conclude that: (i) net-confined fish exhibit hyperthermia; and (ii) this hyperthermia is caused by psychological stress (e.g. anxiety) which they refer to as ‘emotional fever’ [1, p. 1]. Rey *et al*. state that ‘ … lack of emotional fever in fishes … ’ would reflect ‘ … a lack of consciousness … ’ [1, p. 1] and claim that the occurrence of emotional fever in zebrafish ‘ … removes a key argument for lack of consciousness in fishes’ [1, p. 1]. Thus, Rey *et al*. [1] are clearly inferring that their results are consistent with consciousness in fishes. We contend that the methods, analysis and interpretation of their data are flawed and that their conclusions are, therefore, unfounded.

## 1. Conclusion (i)

Rey *et al*.'s [1] conclusion that fish increased their core body temperature is based on a purported shift of the population into warmer chambers following net confinement. However, individual fish could not be identified, and the numerical model used by Rey *et al*. [1] to simulate fish distribution appears to be based on data collected during brief periods that amount to only 1.67% of the total observation time (fig. 1, electronic supplementary material, fig. S2). We used electronic supplementary material, fig. S2, to calculate predicted fish counts in each chamber at time 60 min (table 1). Our analysis suggests that there were only approximately 2 more fish in hyperthermic chambers 5 and 6 compared with controls at any particular moment during the first 4 h post-treatment. These predicted changes in distribution are modest, not statistically significant, and importantly, cannot distinguish between the possibilities that the same fish entered and remained in the hyperthermic chambers versus, for example, whether all experimental fish (or a subset thereof) moved into and out of the hyperthermic chambers. Thus, the analysis presented by Rey *et al*. [1] does not support the inferences made about a stable change in the core body temperature of the fish, let alone their conclusions about stress-induced hyperthermia.

**Table 1.** Predicted fish counts (based on modelling performed by Rey *et al.* [1] in their electronic supplementary material, fig. S2) in each chamber at time 60 min for control and experimental conditions. The column ‘difference in fish count’ represents the change in predicted fish counts in each chamber between experimental and control conditions. Italicized rows are hyperthermic chambers. There is only a modest total increase in predicted fish count of 1.13 (out of a total of approximately 12 fish) in the experimental compared to control hyperthermic chambers. We converted control and experimental fractional values to integers and found no statistical difference between predicted counts in the hyperthermic chambers versus all other chambers (using Fisher's exact test with either one- or two-tailed *p*-values; *p* < 0.05). Hyperthermic chambers in the experimental condition would require a predicted fish count of eight (rather than the current four) to reach statistical significance (*p* < 0.05).

chamber	temperature (°C)	control	experimental	difference in fish count
1	18	0.87	0.08	−0.79
2	25	2.92	1.85	−1.07
3	27	3.06	2.71	−0.35
4	29	2.77	3.17	+0.40
*5*	*32*	*1.89*	*2.65*	*+0.76*
*6*	*35*	*0.94*	*1.31*	*+0.37*
sum		12.45	11.77	−0.68

## 2. Conclusion (ii)

Stress can be triggered by physical stimuli (e.g. injury, pyrogens) and/or psychological states (e.g. emotions). Nonetheless, Rey *et al*. [1] build their case on the unsupported premise that handling and net confinement cause anxiety in zebrafish, which then leads to hyperthermia. While it is not controversial that fish exhibit somatic and physiological responses to stimuli such as net handling [2,3], Rey *et al*. [1] provide no evidence that the purported altered thermal preference by net-confined zebrafish is driven by fish experiencing conscious anxious states. In fact, the idea that stress-induced hyperthermia can be interpreted as an ‘emotional fever’ is highly contentious [4,5], particularly in vertebrate poikilotherms [6,7]. Alternative hypotheses that we contend are more biologically plausible and parsimonious (see below) are not considered by Rey *et al*. [1]. For example, handling of fish causes them to release chemicals (pheromones) into the water that affect cortisol levels in unhandled fish [8]. These pheromones can be released by both very slightly damaged (alarm) and undamaged skin (disturbance substance) during handling and confinement and possibly include factors such as Schreckstoff, urinary ammonia or bile salts [9,10]. Importantly, zebrafish respond to pheromones by changing their swimming behaviour (e.g. more erratic with zig-zagging motions) and their vertical and horizontal position in tanks [11]. Hence, by holding and replacing the net-treated fish back into compartment 3, the release of pheromones into that compartment could explain the purported subsequent change in distribution of these fish. The reported small distribution shift suggests that fish moved towards their preferred normal rearing temperature in chamber 4 and occasionally explored chambers 5 and 6 while avoiding chamber 3 (a behaviour consistent with both conditioned place avoidance and social transfer) [12–14]. Thus, it is just as plausible that Rey *et al.* [1] were measuring chamber ‘avoidance’ rather than chamber preference. In any case, the data provided cannot differentiate between the two alternative explanations.

Because of the incomplete description of methodology, the weak and possibly inappropriate statistical analyses (in particular, inappropriate pooling of dependent samples over time and their analysis by a Mann–Whitney-*U* test for independent samples in their fig. 2), and the high probability that the observations were confounded by experimental artefacts (pheromones), it is impossible to know whether there actually was a shift in the spatial distribution of confined fish or a stable change in the core body temperature of some of the fish, let alone what might have caused those modest purported changes. What is clear is that these results do not support the authors' conclusion that ‘fish can show emotional fever’ [1].

## References

[1] Rey S, Huntingford FA, Boltaña S, Vargas R, Knowles TG, Mackenzie S 2015 Fish can show emotional fever: stress-induced hyperthermia in zebrafish. Proc. R. Soc. B 282, 20152266 (10.1098/rspb.2015.2266) PMC4685827 26609087

[2] Noga EJ, Botts S, Yang MS, Avtalion R 1998 Acute stress causes skin ulceration in striped bass and hybrid bass (*Morone*). Vet. Pathol. 35, 102–107. (10.1177/030098589803500203) 9539363

[3] Ramsay JM, Feist GW, Varga ZM, Westerfield M, Kent ML, Schreck CB 2009 Whole-body cortisol response of zebrafish to acute net handling stress. Aquaculture 297, 157–162. (10.1016/j.aquaculture.2009.08.035) 25587201 PMC4289633

[4] Parviz M, Anushiravani M, Keshavarz M 2013 The relationship between vital spirit and fevers in the ‘Canon of Medicine’: a probable solution for the controversy over stress-induced hyperthermia. Iranian J. Pub. Health 42, 1073–1074. PMC4453889 26060671

[5] Olivier B 2015 Psychogenic fever, functional fever, or psychogenic hyperthermia? Temperature 2, 324–325. (10.1080/23328940.2015.1071701) PMC4843918 27227037

[6] Allen C 2013 Fish cognition and consciousness. J. Agric. Environ. Ethics 26, 25–39. (10.1007/s10806-011-9364-9)

[7] Bovenkerk B, Meijboom FL 2013 Fish welfare in aquaculture: explicating the chain of interactions between science and ethics. J. Agric. Environ. Ethics 26, 41–61. (10.1007/s10806-012-9395-x)

[8] Barcellos LJ, Volpato GL, Barreto RE, Coldebella I, Ferreira D 2011 Chemical communication of handling stress in fish. Physiol. Behav. 103, 372–375. (10.1016/j.physbeh.2011.03.009) 21402090

[9] Ferrari MCO, Wisenden BD, Chivers DP 2010 Chemical ecology of predator–prey interactions in aquatic ecosystems: a review and prospectus. Can. J. Zool. 88, 698–724. (10.1139/Z10-029)

[10] Buchinger TJ, Li W, Johnson NS 2014 Bile salts as semiochemicals in fish. Chem. Senses 39, 647–654. (10.1093/chemse/bju039) 25151152

[11] Speedie N, Gerlai R 2008 Alarm substance induced behavioral responses in zebrafish (*Danio rerio*). Behav. Brain Res. 188, 168–177. (10.1016/j.bbr.2007.10.031) 18054804 PMC2715551

[12] Hall D, Suboski MD 1995 Visual and olfactory stimuli in learned release of alarm reactions by zebra *Danio* fish (*Brachydanio rerio*). Neuro. Learn. Mem. 63, 229–240. (10.1006/nlme.1995.1027) 7670836

[13] Yu L, Tucci V, Kishi S, Zhdanova IV 2006 Cognitive aging in zebrafish. PLoS ONE 1, e14 (10.1371/journal.pone.0000014) 17183640 PMC1762370

[14] Blank M, Guerim LD, Cordeiro RF, Vianna MR 2009 A one-trial inhibitory avoidance task to zebrafish: rapid acquisition of an NMDA-dependent long-term memory. Neuro. Learn. Mem. 92, 529–534. (10.1016/j.nlm.2009.07.001) 19591953

